# Supplementary figures and images for: Topological Analysis of the Type 3 Secretion System Translocon Pore Protein IpaC following Its Native Delivery to the Plasma Membrane during Infection
Source: mBio. 2019 May 28;10(3):e00877-19. doi: 10.1128/mBio.00877-19 (PMC6538787; doi:10.1128/mBio.00877-19)

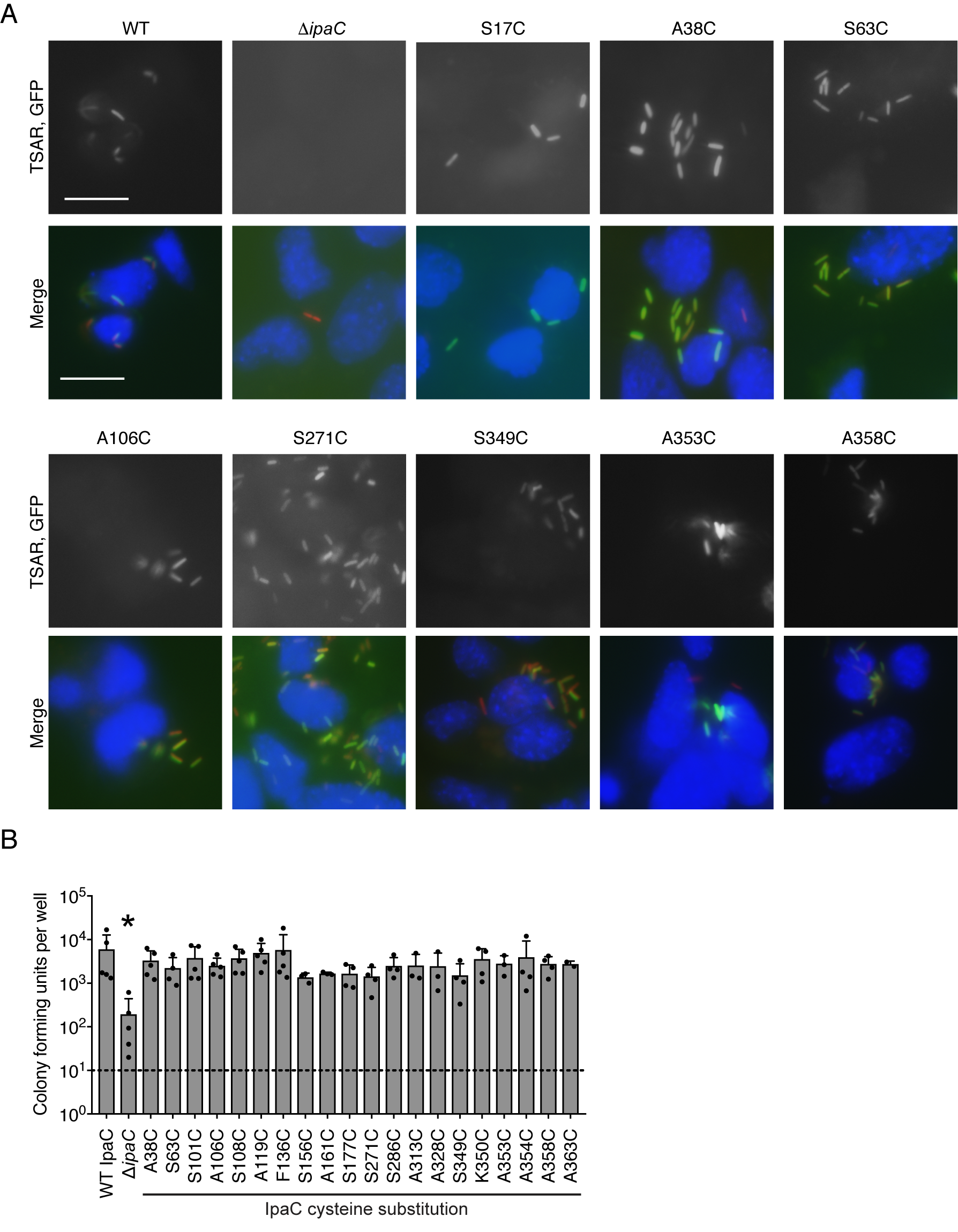

Supplement: FIG S1 [file mBio.00877-19-sf001.tif]

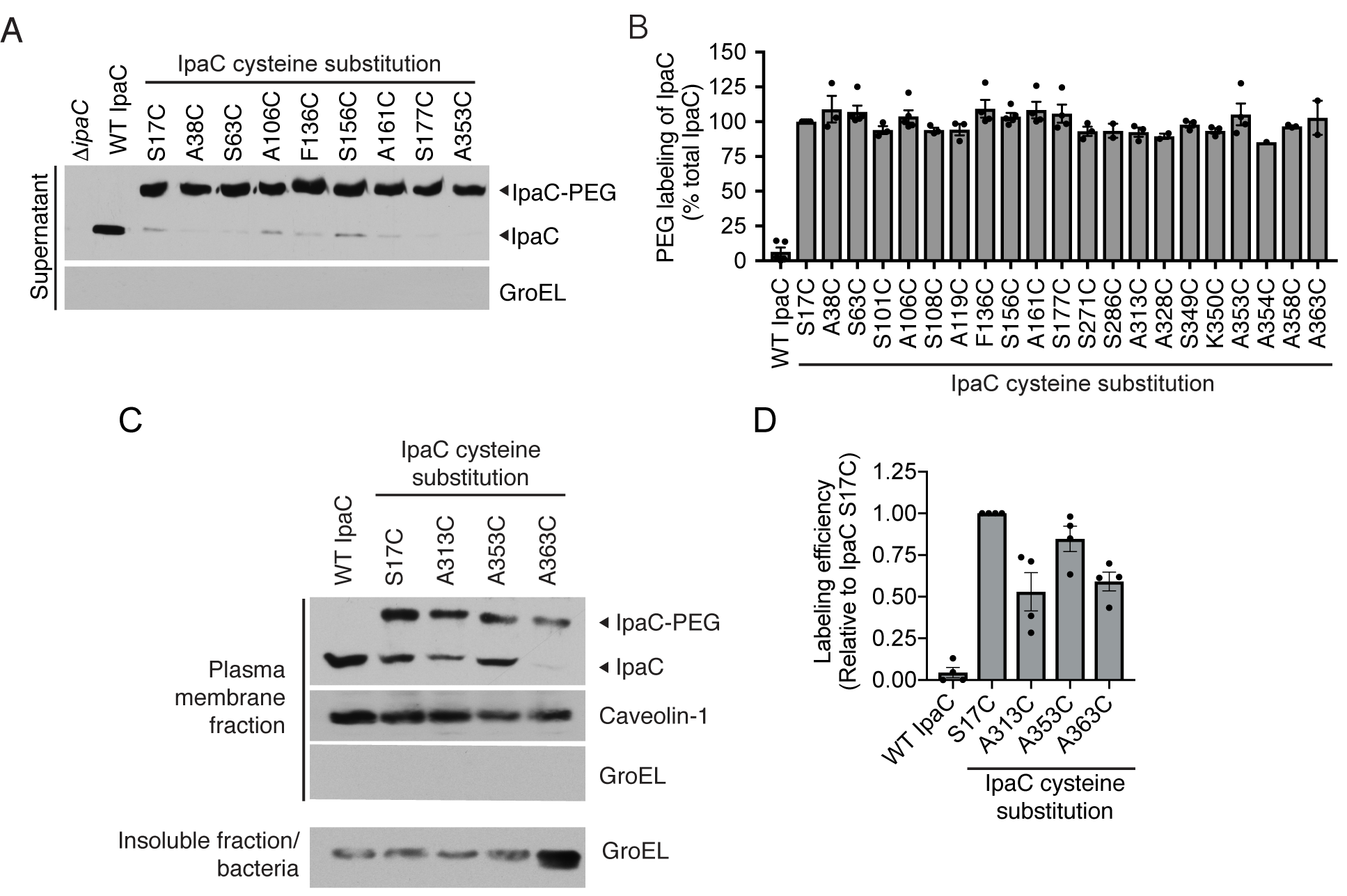

Supplement: FIG S2 [file mBio.00877-19-sf002.tif]

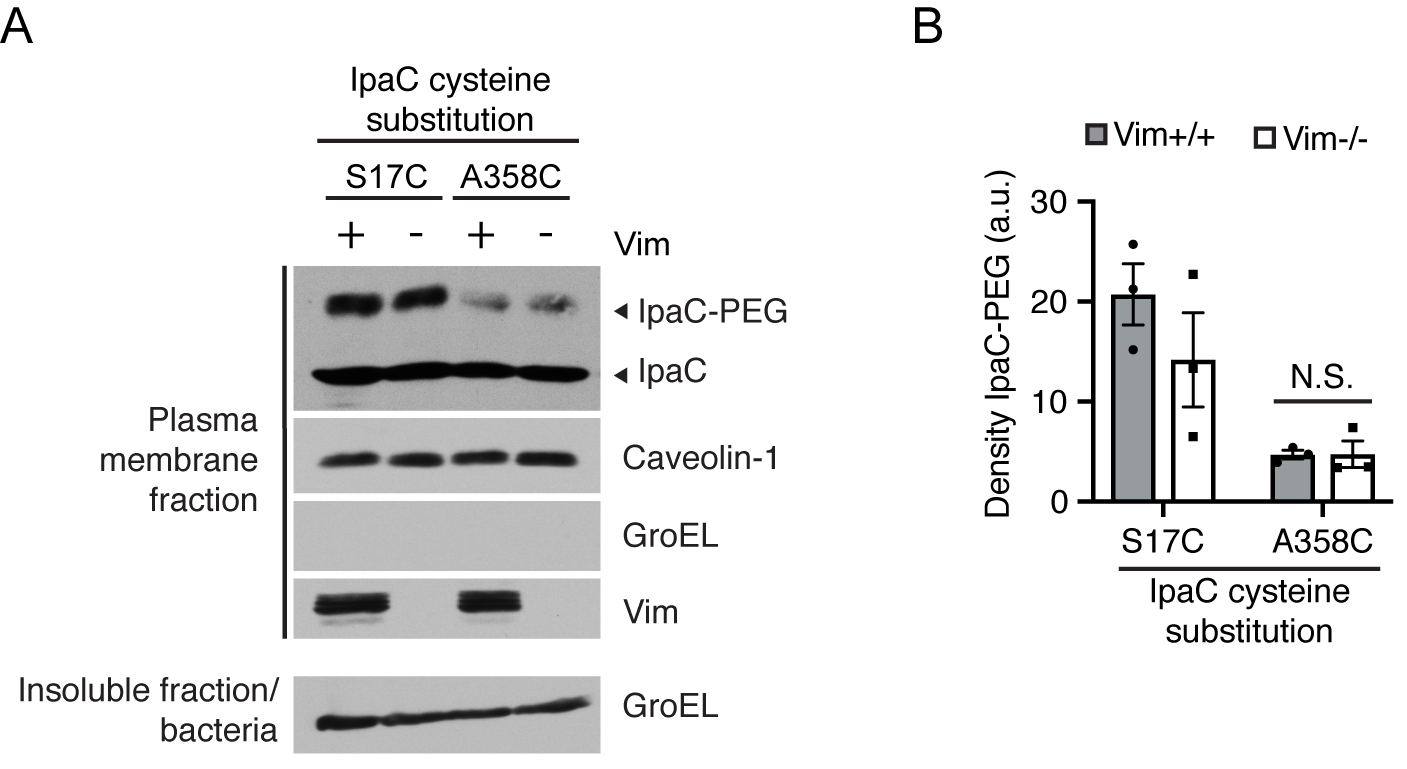

Supplement: FIG S3 [file mBio.00877-19-sf003.tif]

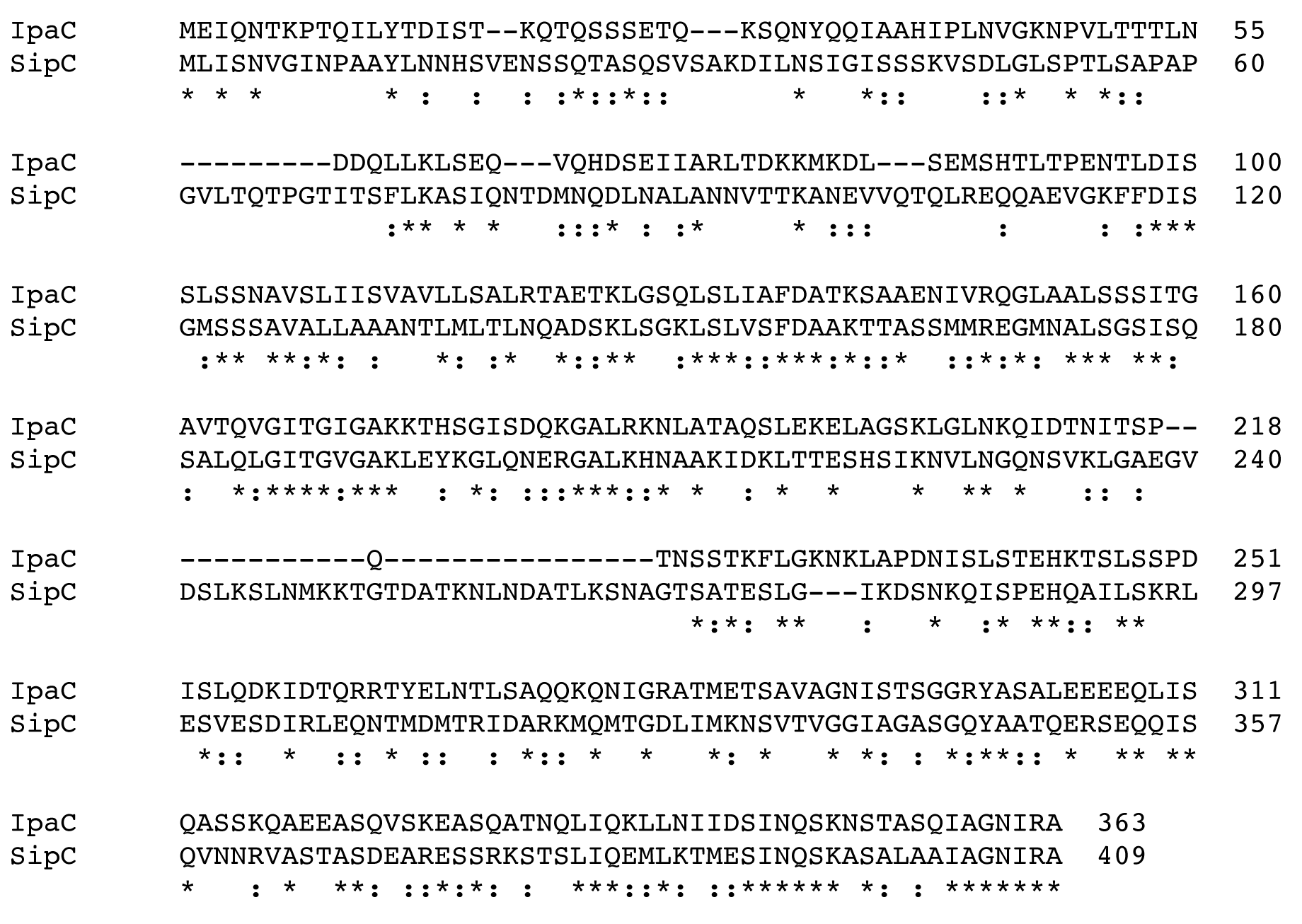

Supplement: FIG S4 [file mBio.00877-19-sf004.tif]

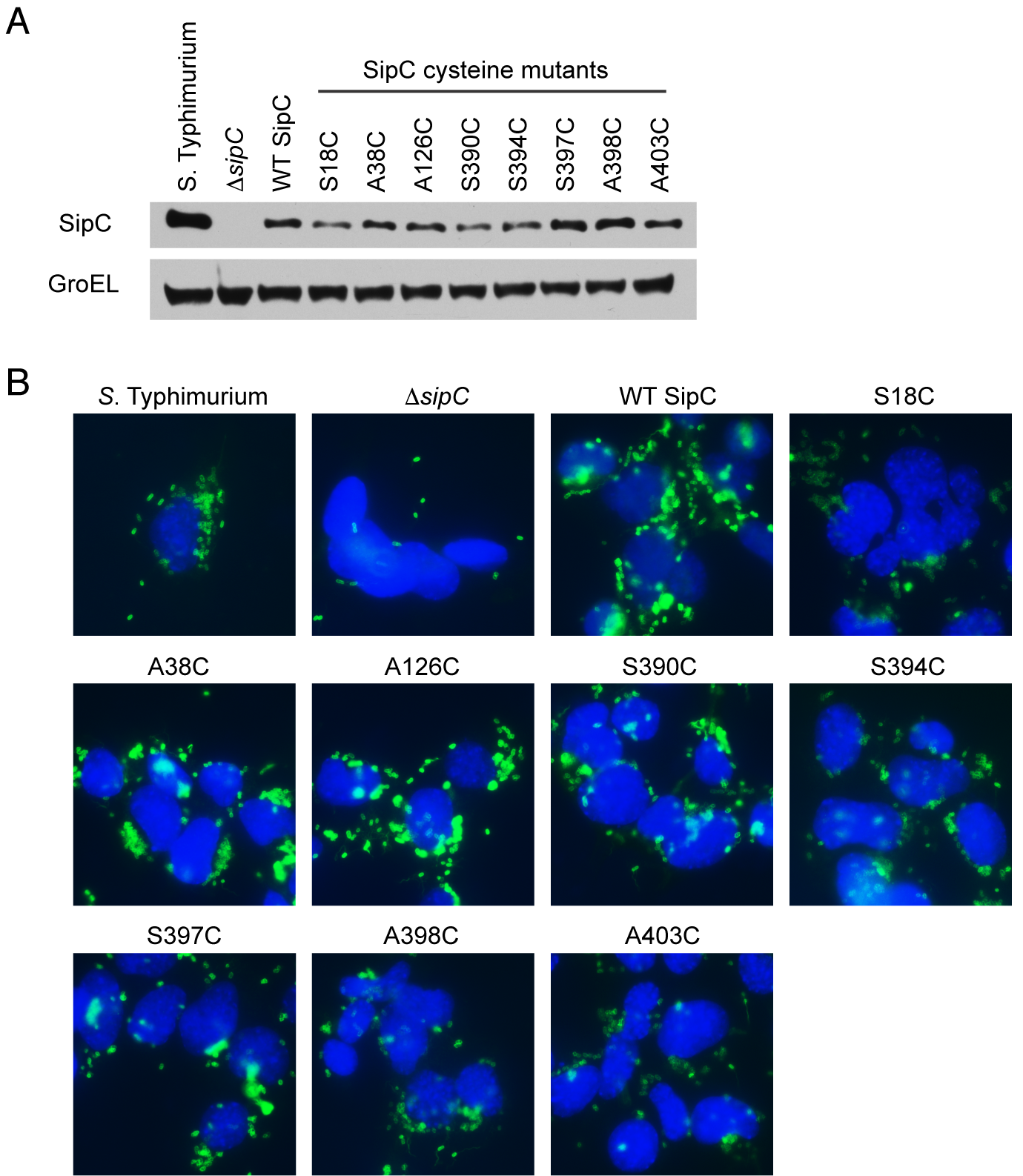

Supplement: FIG S5 [file mBio.00877-19-sf005.tif]
